# PPAR-*γ* Promotes Hematoma Clearance through Haptoglobin-Hemoglobin-CD163 in a Rat Model of Intracerebral Hemorrhage

**DOI:** 10.1155/2018/7646104

**Published:** 2018-07-09

**Authors:** Gaiqing Wang, Tong Li, Shu-na Duan, Liang Dong, Xin-gang Sun, Fang Xue

**Affiliations:** ^1^Department of Neurology, Shanxi Medical University, 56 Xinjian S Rd., Yingze, Taiyuan, Shanxi 030001, China; ^2^Department of Neurology, The Second Hospital, Shanxi Medical University, 382 WuYi St., Taiyuan, Shanxi 030001, China

## Abstract

**Background and Purpose:**

PPAR-*γ* is a transcriptional factor which is associated with promoting hematoma clearance and reducing neurological dysfunction after intracerebral hemorrhage (ICH). Haptoglobin- (Hp-) hemoglobin- (Hb-) CD163 acts as a main pathway to Hb scavenging after ICH. The effect of PPAR-*γ* on the Hp-Hb-CD163 signaling pathway has not been reported. We hypothesized that PPAR-*γ* might protect against ICH-induced neuronal injury via activating the Hp-Hb-CD163 pathway in a rat ICH model.

**Methods:**

107 Sprague-Dawley rats were used in this research. They were randomly allocated to 4 groups as follows: sham group, vehicle group, monascin-treated group, and Glivec-treated group. Animals were euthanized at 3 days after the model was established successfully. We observed the effects of PPAR-*γ* on the brain water content, hemoglobin levels, and the expressions of CD163 and Hp in Western blot and real-ime PCR; meanwhile, we measured hematoma volumes and edema areas by MRI scanning.

**Result:**

The results showed that PPAR-*γ* agonist significantly reduced hematoma volume, brain edema, and hemoglobin after ICH. It also enhanced CD163 and Hp expression while PPAR-*γ* antagonist had the opposite effects.

**Conclusions:**

PPAR-*γ* promotes hematoma clearance and plays a protective role through the Hp-Hb-CD163 pathway in a rat collagenase infusion ICH model.

## 1. Introduction

In Western societies, intracerebral hemorrhage (ICH) takes up for 8–15% of all strokes and 20–30% in the Asian area, and there is no definite effective therapy so far [1, 2]. Understanding the complex pathophysiology of cerebral injury after ICH is crucial to developing new approaches to reduce the harmful impacts on ICH.

The occurrence of ICH begins with a vast release of blood within the brain parenchyma [3, 4]. Erythrocytes, as the major cellular components of the hematoma, dissolves and releases hemoglobin (Hb) which subsequently broke down into heme and iron after ICH within a few days [5]. These cytotoxins mainly cause secondary brain injury following ICH [6]. Haptoglobin-Hb-CD163 as well as hemopexin-heme-LRP1 (low-density lipoprotein receptor-related protein-1) is believed to be the most important endogenous scavenging pathway which participates in hematoma/blood component resolution following ICH [6]. The cell-free Hb can trigger oxidative damages caspase activation, blood-brain barrier disruption, and neuronal death and result in irreversible brain damages [7]. CD163, which is the only hemoglobin clearance receptor expressed in the mononuclear phagocyte system, is formed during the hemolysis of erythrocytes and mediates the endocytosis of the Hb, leading to the degradation of the ligand protein and cytoplasmic heme oxygenase [8]. Haptoglobin (Hp), which is a primary Hb-binding protein, attenuates the destructive effects of Hb in the plasma [6, 9, 10]. Superabundant Hb in the plasma can upregulate the expression of Hp and the Hb-Hp receptor CD163 in neurons [11]. Hp is bound to free Hb and once Hp-Hb complex is endocytosed by CD163 may cause an anti-inflammatory response. The Hp-Hb-CD163 acts as the main pathway in Hb scavenging and exerts a pivotal protective role [9, 12].

PPAR-*γ* is a transcription factor which can regulate the expression of catalase and superoxide dismutase which are two important antioxidant genes [13, 14]. It is also associated with promoting hematoma clearance and reducing neurological dysfunction [15]. As a PPAR-*γ* agonist, monascin is the main component of red yeast rice with a Chinese traditional technique and has been shown to have a protective effect by promoting hematoma clearance and reducing cerebral edema in rats after ICH [13], but the specific mechanism of monascin in ICH has not been clarified so far.

We hypothesize that PPAR-*γ* will promote hematoma clearance via CD163 and Hp upregulation, therefore reducing brain edema and improving BBB integrity after ICH. So we designed the study to test the effect of PPAR-*γ* on the Hp-Hb-CD163 pathway through PPAR-*γ* agonist monascin and its antagonist Glivec which mediates PPAR-*γ* by declining the phosphorylation level [16] in a collagenase-induced ICH rat model.

## 2. Materials and Methods

### 2.1. Animal Preparation

This study used 107 male adult Sprague-Dawley rats, weighing about 250~300 g (from Shanxi Medical University Animal Laboratory). The protocol for using these animals was in accordance with the Animal Utilization and Management Committee which was made by Shanxi Medical University. All rats were available to get fodder and water freely in the research.

### 2.2. Animal Treatments and Experimental and Control Groups

All rats were randomized to the following groups: sham operation group (*n* = 25), vehicle group (*n* = 27), monascin-treated group (10 mg/kg twice a day, *n* = 26), and Glivec-treated group (100 mg/kg/day, *n* = 29). Dead animals were replaced before final assessment. All gavages were administered by gastric perfusion 6 h after ICH until the endpoint.

### 2.3. Intracerebral Hemorrhage Model of Rats

The intracerebral hemorrhage model was made by injecting collagenase IV to the corpus striatum under a head stereotaxic apparatus [13]. Briefly, experimental rats were anesthetized by hydrated chloric aldehyde (300–350 mg/kg) in an intraperitoneal injection method. After being anesthetized, rats were positioned in the stereotactic instrument (Jiangwan type 1 C Instrument, Shanghai, China). A 1 mm needle was inserted through a cranial burr hole into the striatum to the following frame of references: 0.5 0mm anterior, 5.8 mm ventral, and 2.3 mm lateral to the bregma. Then, we used a 5 *μ*L flat-headed microsyringe (Hamilton 600, Switzerland) to infuse 0.5 U type IV collagenase (Sigma-Aldrich, USA) which was dissolved in 2.5 *μ*L saline solution. After infusion, the needle needs to be maintained in there for extra 3 minutes and subsequently be pulled out slowly. In the sham group, 2.5 *μ*L saline solution was infused using the same method. After the surgery, the hole in the skull was sealed and the scalp was well sutured. Animals were bred in a specific facility which was pathogen free. Besides, they can get food and water uncontrolled.

### 2.4. Brain Water Content

The water content of rat brain tissue was performed as earlier described [13]. We used 4% chloral hydrate for intraperitoneal injection to deeply anesthetize the rat, and then the rat was decapitated to measure the cerebral water content. The brain tissue was removed from the skull rapidly and then divided into 4 mm sections in the portion around the puncture point. All brain tissue samples we got from the ipsilateral basal ganglia were instantly weighed by an electric microbalance to know the wet weight (Ww). Then tissues were placed in a 100°C drying oven for 48 hours to desiccation. After that, we can obtain dry weight (Dw). The brain water content was calculated by the following formula: (Ww − Dw)/Ww × 100%.

### 2.5. Hemoglobin Assay

Quantitation of brain hemoglobin after ICH was measured by hemoglobin assay under the guidance of the manufacturer's instructions. Briefly, successful modeling rats were sacrificed and the brain tissues were quickly removed and put into four glass dishes, respectively. A total of 1000 *μ*L prerefrigerated PBS buffer was added into each glass dish. Brain tissue was smashed by sonication, collected in a centrifuged tube, and centrifuged at 4°C, 12000 rpm for 30 minutes. 25 *μ*L of the supernatant of each group was put into a 96-well plate, and Drabkin's reagent was added to the supernatant in a ratio of 1 : 4. After incubation for 5 minutes at room temperature, OD value was measured by a spectrophotometer in 400 nm. The OD value of each sample was calibrated by a blank group.

### 2.6. Expression of PPAR-*γ*, CD163, and Hp in Different Groups by Western Blot

The brain tissue was smashed, and RIPA Lysis Buffer with PMSF was added for extracted total protein in each sample for Western blot analysis. Protein concentration was determined by a bicinchoninic acid (BCA) assay. 50 *μ*g of each sample lysis was loaded on a 10% sodium dodecyl sulfate gel and electrophoresed in 90 volts for 2 hours. Belt was transferred to the polyvinylidene fluoride membrane after an electrophoresis process. Membranes were blocked with 5% BSA blocking buffer at 37°C for 2 hours and incubated with first antibodies: polyclonal anti-PPAR-*γ* of rabbit (1 : 1000, Bioss), anti-CD163 of rabbit (1 : 500, Bioss), and polyclonal anti-Hp of rabbit (1 : 500, Bioss) at 4°C in a thermostat shaker overnight. Meanwhile, other membranes were probed with *β*-actin (1 : 3000, Bioworld) as an internal control. After being washed by the TBST buffer, all membranes were incubated with the second antibodies at 37°C for 2 hours. Immunoreactive membranes were processed with an ECL Plus chemiluminescence assay kit. After that, it can be visualized through an imaging system (Bio-Rad, ChemiDoc). Finally, band intensities were normalizing with their internal controls, respectively, and digitizing using ImageJ software.

### 2.7. Measurements of Volume of Hematoma and Cerebral Edema by MRI

All rats were given brain MRI scan on a 1.5 T clinical scanner (GE Signa HDx, GE healthcare Milwaukee) with a knee coil 3 days post-ICH at the Second Hospital affiliated to Shanxi Medical University. During the MRI imaging scanning, rats were maintained well anesthetized after the use of 5% chloral hydrate with the prone position. A series of MR sequences were acquired in our study, the protocol included T2-weighted imaging (T2WI) and T2 Flair to assess the edema, and scanning parameters [13] are listed as follows: repetition time (TR)/echo time (TE) = 2400/129 ms, field of view (FOV) = 18 × 18 mm, slice thickness = 2.0 mm, matrix size = 512 × 448, and interval = 0.2 mm. In T2 fluid-attenuated inversion recovery (T2 Flair), TR/TE = 8502/128.6 ms, FOV = 12 × 12 mm, slice thickness = 2.0 mm, matrix size = 512 × 448, and interval = 0.2 mm. T2^∗^-weighted imaging (T2^∗^WI) and susceptibility weighted imaging (SWI) were used to determine the hematoma size; scan parameters are as follows: in T2^∗^WI, TR/TE = 400/15 ms, FOV = 18 × 18 mm, slice thickness = 2.0 mm, matrix size = 448 × 384, interval = 0.2 mm, and flip angle = 15°. In SWI: TR/TE = 49.9/4.5 ms, FOV = 18 × 18 mm, slice thickness = 1.5 mm, flip angle = 15°, and matrix size = 448 × 448. MRI postprocessing was performed on an off-line workstation by two experienced neurologists who were blinded to the group set and scan date. The absolute volume of intracerebral hemorrhage area which contains the outer amount of edema and hematoma was adopted during the measurement process. The total value of the absolute volume was calculated by integrating injured areas of brain hemorrhage slices. All the assessments were repeated three times, respectively. The results were shown as the mean and standard deviation.

### 2.8. Assay of Haptoglobin and CD163 in Different Groups by Real-Time PCR

The total RNA of different groups was extracted from the brain tissue surrounding hematoma by using TRIzol Reagent (Takara Inc., Japan) complied with the manufacturer's instructions. After completing the extraction process, total RNA was determined by Nano-drop 2000 (Thermo Fisher, USA) with the UV absorbance at 260 nm to ensure purity. Complementary DNA was reverse transcribed by using a one-step PrimeScript™ RT Master Mix kit (Takara Inc., Japan), and a total of 20 *μ*L reaction mixture system which contained 1 *μ*g total RNA was carried out at 37°C for 15 minutes; finally, the complementary DNA was kept at a minus 80°C environment. Real-time PCR analysis was processed in a BIO-RAD iCycler Thermal Cycler for RT-PCR (Bio-rad, USA) with the complementary DNA and SYBR® Premix Ex Taq™ Kit (Takara Inc., Japan). Oligonucleotide PCR-based primers are as follows: haptoglobin: 5′-gaaaggcgctgtaagtcctg-3′ (forward primer) and 5′-tcctcttccagggtgaattg-3′(reverse primer) and CD163: 5′-gacagacccaacggcttaca-3′ (forward primer) and 5′-ggtcacaaaacttcaaccgga-3′(reverse primer). The experiment uses a 25 *μ*L volume total reaction mixture reaction system which contains 2 *μ*L of the diluted complementary DNA product, 12.5 *μ*L of the SYBR Premix Ex Taq Mix (Takara Inc., Japan), 1 *μ*L of forward/reverse primers, respectively, and 8.5 *μ*L of RNase-free water. The condition of real-time PCR reaction was implemented as follows: predenaturation step was processed at 95°C for 1 minute. The extended process sets the denaturation at 95°C for 30 seconds and annealing and elongation at 55°C for 45 seconds, and the extended process was repeated for 40 cycles. Reverse transcription PCR was performed three times for each sample. To standardize the expression of haptoglobin and CD163 mRNA, the levels of the reference gene *β*-actin were determined for each sample parallelly. Expression of final results was ratios of the target gene copy numbers to *β*-actin transcripts. The expression of the targeted gene was computed by the 2^−ΔΔCt^ method.

### 2.9. Statistical Analysis

Quantitative data were sorted out as the mean ± SD. One-way ANOVA was taken for multiple comparisons. The SNK-q test was adopted for the comparison of the differences between groups of brain water content, hemoglobin levels, and real-time PCR assay, while the differences of MRI parameter and Western blot results were determined by Tukey's post hoc test. *p* < 0.05 was denoted the difference processing statistical significance among all groups.

## 3. Results

### 3.1. Mortality

The overall mortality in operative rats was approximately 10.2% (*n* = 11). All the sham group rats survived, and there was no significant difference in the mortality of each group (data not shown).

### 3.2. PPAR-*γ* Agonist Monascin Decreased Brain Water Content

**Figure 1:**
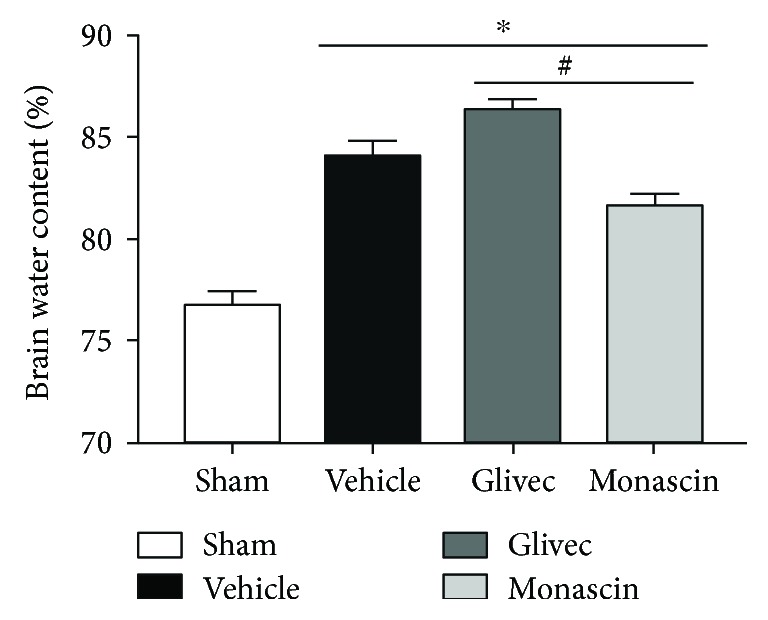
Effect of PPAR-*γ* on brain water content associated with ICH 3 days after surgery (^∗^*p* < 0.05 versus sham; ^#^*p* < 0.05 versus vehicle).

All the operative groups showed a significant increase in brain water content when compared to the sham group (^∗^*p* < 0.05 versus sham; Figure 1). PPAR-*γ* agonist monascin significantly lowed the water content of brain tissue around hematoma while PPAR-*γ* antagonist Glivec acted the opposite way, in comparison with the vehicle group (^#^*p* < 0.05; Figure 1).

### 3.3. PPAR-*γ* Agonist Monascin Reduced Hemoglobin Level

**Figure 2:**
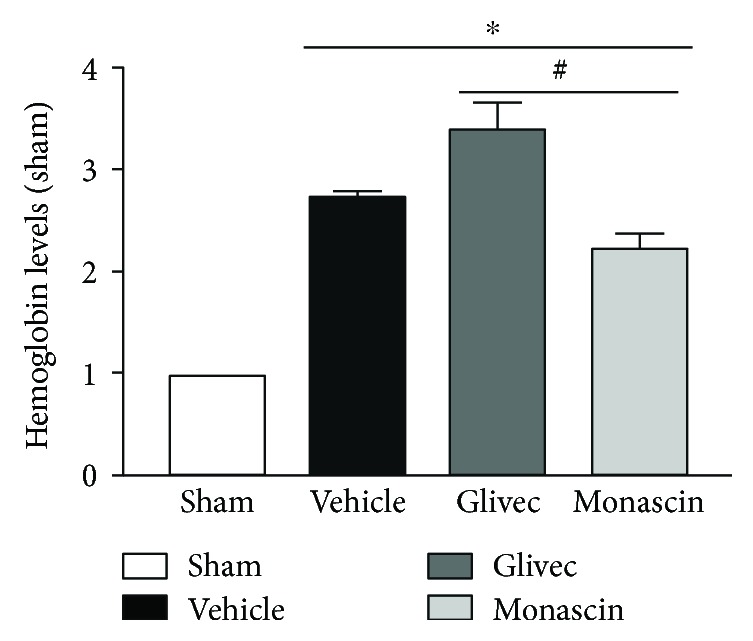
Effect of PPAR-*γ* on hemoglobin levels associated with ICH 3 days after surgery (^∗^*p* < 0.05 versus sham; ^#^*p* < 0.05 versus vehicle).

The hemoglobin level of all the operative groups was obviously higher than that of the sham group (^∗^*p* < 0.05; Figure 2). Compared to the vehicle group, monascin significantly decreased the level of hemoglobin (^#^*p* < 0.05 versus vehicle), while Glivec increased it (^#^*p* < 0.05 versus vehicle).

### 3.4. Effect of Monascin and Glivec on CD163 and Hp Expression following ICH

**Figure 3:**
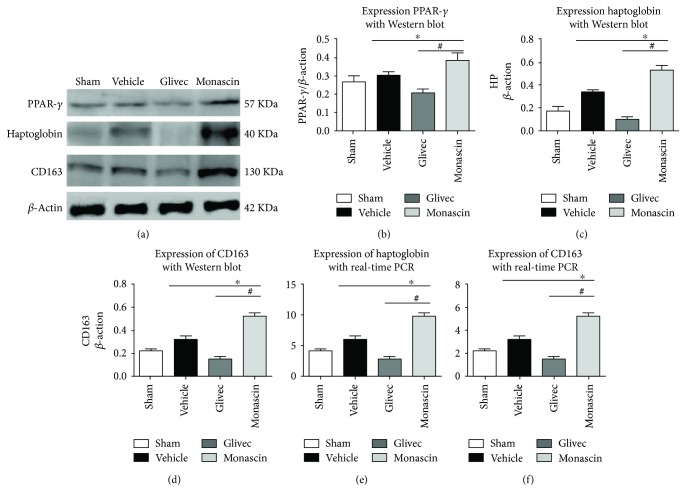
Effect of Glivec and monascin on PPAR-*γ*, haptoglobin, and CD163 associated with ICH 3 days after surgery. Representative images are shown of Western blot assay (a–d) and real-time PCR (e and f) for PPAR-*γ*, haptoglobin, and CD163 levels within ipsilateral brain tissues. One-way ANOVA followed by Tukey's tests was used. (^∗^*p* < 0.05 versus sham; ^#^*p* < 0.05 versus vehicle).

The results of Western blot and PCR showed a significant increase in PPAR-*γ*, Hp, and CD163 expression within ipsilateral brain tissues after ICH when compared to sham (^∗^*p* < 0.05, Figure 3). Compared to vehicle, monascin increased PPAR-*γ*, Hp, and CD163 expression with Western blot (^#^*p* < 0.05, Figures 3(a)–3(d)) and real-time PCR (^#^*p* < 0.05, Figures 3(e) and 3(f)). Meanwhile, the administration of Glivec downregulated the expression of PPAR-*γ*, Hp, and CD163 (^#^*p* < 0.05, Figure 3).

### 3.5. Monascin Decreased the Volume of Hematoma (T2^∗^WI/SWI) and Brain Edema (T2WI/T2 Flair) in the Rat Model after ICH

**Figure 4:**
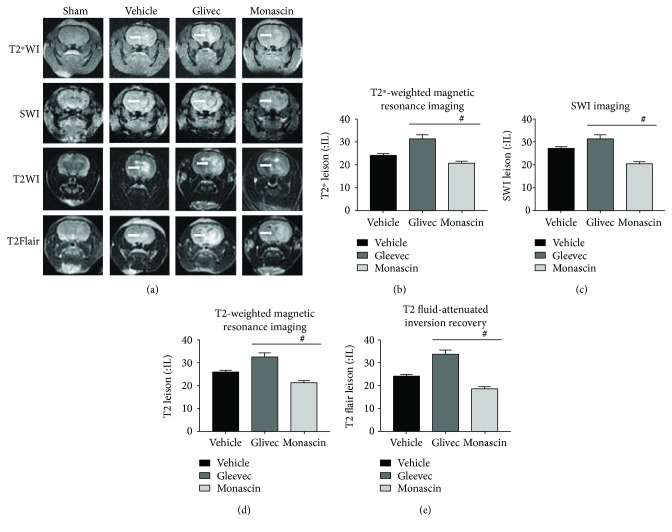
Effect of PPAR-*γ* on hematoma volume (a–c) and brain edema (a, d, and e) associated with ICH 3 days after surgery. Representative images are shown of T2^∗^WI (a and b), SWI (a and c) for hematoma volume, T2WI (a and d), and T2 Flair (a and e) for brain edema within ipsilateral brain tissues. One-way ANOVA followed by Tukey's tests were used (^#^*p* < 0.05 versus vehicle).

The volumes of hematoma and edema of all groups were measured at 3 days after modeling successfully (showed in Figure 4). The volume of hematoma and edema was reduced in the monascin group compared to the vehicle group. While Glivec extended the volume of hematoma and edema 3 days after ICH. The link assay between brain edema and hematoma lesion showed a positive correlation between them (*r* = 0.989, *p* = 0.011).

## 4. Discussion

In our study, we demonstrated that PPAR-*γ* is neuroprotective through decreasing hematoma size and hemoglobin levels then reduced brain edema. PPAR- *γ* agonist monascin enhanced haptoglobin and CD163 expression whereas PPAR-*γ* antagonist Glivec had the opposite effects on a rat ICH model.

Intracerebral hemorrhage is a devastating disease, and there has been no specific therapy to reduce the mortality [17]. It started from the blood's massive release into the brain parenchyma [3, 11, 18]. The red blood cell (RBC) lyses within several days and releases Hb at the same time [6]. The hematoma is the culprit of brain insults after ICH, so how to effectively remove blood products is crucial in ICH-induced brain injury [19].

Hp is a glycoprotein which is abundant in the plasma [20]. It is mainly secreted by hepatocytes, and a mononuclear phagocyte system can also produce it [21]. The levels of Hp in the plasma increases to answer stress response and anti-inflammation, which bond to free Hb after cerebral hemorrhage [14, 19]. The formation of Hp-Hb complex protects Hb from oxidative modifications. Otherwise, oxidative modification can prevent the clearance processing and lead the releasing of free Hb into the circulation of the blood [22]. Besides, the Hp-Hb-CD163 complex has a high-affinity site for CD163 to recognize and promote hemoglobin clearance [8, 9, 14].

CD163 acts as a hemoglobin scavenger receptor. It is only expressed in the monocyte-macrophage system [9] and is a 130 kDa transmembrane glycoprotein which can be combined with a variety of ligands. It also belongs to scavenger receptor superfamily class B [18]. CD163 is the cellular receptor target of Hp after ICH [10]. After recognization by the Hp-Hb complex, the Hp-Hb-CD163 complex system is formed during the hemolysis of erythrocytes and mediates the endocytosis of the hemoglobin, leading to the degradation of the lysosomal ligand protein [8]. The Hp-Hb-CD163 acts as the main pathway in Hb scavenging and exerts a pivotal protective role [9].

PPAR-*γ* is a transcription factor belonging to the nuclear hormone receptor superfamily. During the past years, the transcription factors of PPAR-*γ* [19, 23] were validated as important players in regulating phagocyte-mediated cleanup processes and able to promote endogenous hematoma absorption, decrease neuronal damage, and improve functional recovery in a rodent model of ICH [24]. It not only increased microglia-mediated phagocytosis of RBC in rat primary microglia in culture but also reduced the generation of peroxide during the phagocytic process [25]. The specific mechanism of PPAR-*γ* in ICH has not been completely clarified so far.

In the present study, we found that PPAR-*γ* agonist monascin is neuroprotective by decreasing the brain water content and the level of hemoglobin. Besides, it also enhanced CD163 and Hp expression in Western blot and real-time PCR results whereas Glivec reduced Hp and CD163 expression.

Magnetic resonance imaging (MRI) is a medical imaging technique and has been extensively used in the study of intracerebral hemorrhage [14]. It has high sensitivity for presenting the temporal and spatial shifts of hematoma and edema after ICH. At 3 days after surgery, we assessed the volume of hematoma and cerebral edema via T2^∗^WI/SWI and T2WI/T2 FLAIR sequences [7]. The results showed PPAR-*γ* agonist monascin evidently reduced hematoma volume and cerebral edema after ICH, while the Glivec expanded the hematoma and edema areas.

Our results demonstrated that PPAR-*γ* agonist monascin decreased hematoma volume and brain edema in a collagenase-induced ICH rat model via histology, molecular biology, and MRI imaging methods. Meanwhile, monascin upregulated the expression of CD163 and Hp which belong to the endogenous hemoglobin scavenging system in ICH.

PPAR-*γ* activation reinforced microglia-induced erythrocyte phagocytosis. Our previous study demonstrated that PPAR-*γ* agonist improved outcome through reducing hematoma volume and edema formation following ICH [13]. While macrophages play a central role in hematoma clearance, hemoglobin mostly remains encapsulated within erythrocytes until they are phagocytosed and degraded by microglia and infiltrating macrophages [1]. CD163, a hemoglobin scavenger receptor, is mainly expressed on macrophages/microglia, and it plays a major role in scavenging free hemoglobin released during erythrolysis after ICH. CD163 transports hemoglobin into microglia/macrophages and functions as a membrane-bound scavenger receptor for clearing extracellular haptoglobin-hemoglobin (Hp-Hb) complexes [11]. Excessive Hb upregulated the expression of Hp and the Hb/Hp receptor CD163 in vivo and in vitro. Free Hb binds to Hp and once Hp-Hb complex is endocytosed by CD163, which mediated the delivery of Hb to the macrophage, may fuel an anti-inflammatory response because heme metabolites have potent anti-inflammatory effects [6]. So PPAR-*γ* activation possibly reinforced microglia-induced Hp-Hb complex phagocytosis through enhancing CD163 expression.

In conclusion, PPAR-*γ* promotes hematoma clearance and plays a protective role possibly through the Hp-Hb-CD163 pathway in a rat collagenase-induced ICH model. Monascin, as a PPAR-*γ* agonist, will be a potential medical treatment for ICH in the future.

## Data Availability

The data used to support the findings of this study are available from the corresponding author upon request.
